# Dental care in children with Down syndrome: A questionnaire for Belgian dentists

**DOI:** 10.4317/medoral.22129

**Published:** 2019-04-24

**Authors:** Isabelle Descamps, Carla Fernandez, Diane Van Cleynenbreugel, Yann Van Hoecke, Luc Marks

**Affiliations:** 1 Centre of Special Care in Dentistry, Ghent University Hospital, Belgium; 2 Chambres Syndicales Dentaires, Brussels, Belgium; 3 Flemish professional organisation for dentists (VBT), Ghent, Belgium

## Abstract

**Background:**

To date, research on the opinions of dentists on the oral health care of children with DS (Down Syndrome) is scarce.

**Material and Methods:**

Evaluate the views and knowledge of Belgium dentists regarding dental care of children with DS. An adequate sample of dentists were invited to fill in a validated questionnaire. Results were assessed in 95% confidence interval with *p*< 0.05 level.

**Results:**

A total of 356 questionnaires were returned (177 men, 179 women). Mean age of the dentists was 50.3 years (SD: 11.9) and 75% obtained their degree more than 20 years ago. 72.5% of all dentists replied that they had not been instructed in how to treat children with DS during their dental educational training, whereas this is only the case for 39% of the dentists who obtained their degree less than 10 years ago. Half of the group indicated that additional training and education would be (very) desirable (52.8%).

**Conclusions:**

Dentists don’t seem to feel comfortable in treating children with DS and refer them to a special care dentistry centre in a hospital. It is positive that dentists are in favour of obtaining additional training and education to help them feel more confident in treating children with DS in daily practice. However we must not conclude that because students or qualified dentists received such training that they will automatically treat more patients with special needs.

** Key words:**Oral health care, Down syndrome, children, dentists, Belgium.

## Introduction

It’s known that the risks for children with DS suffering from numerous medical problems has increased. Congenital heart diseases, visual impairment, hearing problems, neurological and immunological deficiencies and gastrointestinal tract problems are the most common (1). Parents are therefore recommended to visit a physician on a regular basis to monitor and prevent those medical problems (2). Patients with DS are also affected by various intra and extra oral problems. Anatomical differences in the middle third of the face, in combination with developmental differences, such as hypotonia of orofacial muscles, can cause functional problems including breastfeeding, swallowing, chewing and speaking problems. (3,4) Children with DS tend to protrude their tongue, due to the hypotonic orofacial muscles. In order to obtain a more stable occlusion, they also protrude their mandible. The combination of tongue thrusting and a prognathic mandible can lead to open mouth breathing which can be a trigger for Obstructive Sleep Apnea Syndrome (OSAS) and airway infections. (4,5) Due to this open mouth breathing the accumulation of plaque is increased and the natural cleansing mechanism of the saliva is disturbed.

Tooth agenesis, hypoplasia and hypo-calcification are regularly seen (3). Whether children with DS are at higher risk for caries, is still a point of discussion in the literature (3,4). Delayed tooth eruption, microdontia and spacing or tooth agenesis are possible reasons for a lower caries risk in children with DS (3,4). The disturbed cleansing mechanism of saliva and the presence of interdental food residues can cause a lower oral pH value. Therefore demineralisation and a higher prevalence of caries can occur (4,5). In the study of Loureiro *et al.* it was shown that gingivitis was found in 91% of the children with DS aged between 6 and 20 years old. Attachment loss due to periodontitis was found in 36% of the children younger than 6 and 94% of the children between 16 and 20 years old were diagnosed to have an aggressive form of periodontitis (6).

In view of these observations, the role of dentists is crucial in the observation, examination, treatment and follow up of children/ patients with DS. A recent study of the National Health Insurance Institute in Belgium showed that parents of children with special health care needs still experience problems in accessing dental care (7). These barriers are for example financial problems, transport to the dentist but also psychological or sociological problems. The dentist also needs to be giving proper information about oral problems, development and oral health care on an individual level.

A questionnaire or survey would be a valuable tool to obtain information regarding dentists’ knowledge concerning the oral health of DS patients. A result of the shift in the educational programme during the past decade where undergraduate students are normally more exposed to patients with disabilities, should be seen. In order to be representative of Belgium as a whole, a questionnaire should be available in both Dutch and French as these are the official languages spoken by the majority of the population. Because of the political differences between parts of the country (North/Flanders and South/Wallonia), an overall result should be obtained by conducting the questionnaire in both parts of Belgium.

For this study we focused on the following topics 1) What is the experience of general dentists in treating children with DS?; 2) What are the barriers that general dentists may experience in the treatment of children with DS?; 3) What did they learn about the treatment of children with DS during their educational program?

## Material and Methods

A questionnaire was developed, partially based on questionnaires from the literature (8,9). This questionnaire was distributed during major dental meetings in Flanders and in Wallonia where dentists were invited to fill in the questions. Both meetings were meant for general dentists with subjects like prosthetics, aesthetic dentistry, periodontics and orthodontics. The questionnaires were collected immediately. As stated on the attached letter, participation was voluntary and anonymous and by filling in the questions, permission was given to use the data.

The questionnaire was written in Dutch and translated into French to obtain data from the dentists of the French-speaking part of Belgium. This French questionnaire was also retranslated into Dutch to test the validity. The first draft was proof read by 5 dentists and 7 non-dentists. The feedback of this test panel was taken into account and adaptations were made to ensure that questions and terms were clear. Study approval (including data protection) was obtained from the Ethics Committee of Ghent University Hospital (B670201524264, EC UZG 2015/0296) according to the Helsinki convention.

The survey comprised two sections. In the first part, general and demographic information of the dentist and his/her dental practice was collected. The second part contained closed questions in different categories: How many children with or without special needs or DS does the dentist treat in one week? A five point Likert scale was used (very often-often-sometimes-rarely-never). How confident does the dentist feel about treating children with DS and did s/he get training for this during their dental education? A 6 point-Likert scale was used (not at all-not really-neutral-well-very well-no opinion) (8,9). Does the dentist require any additional training and what are the barriers to treating children with DS? The possible barriers were rated on a four point Likert scale (high-medium-low-no barrier- not applicable) (8). Is their dental office and waiting room accessible for patients with special needs (mental and/or physical disabilities)? This question was answered with a value between 0 (not accessible at all) and 10 (very accessible) (10). What kind of anaesthesia or sedation does the dentist use? Who is, in the opinion of the dentist, the right dentist to treat children with DS (general dentist, paediatric dentist, special care dentist)? Dentists were also asked to assess their competencies in giving information to parents about oral problems associated with DS and performing different dental procedures in children with DS (11).

The data were systematically collected and missing data were noted.

Dentists had to fill in all parts of the questionnaire and questionnaires had to be filled in as completely as possible. When 80% of the questions were not filled in, the questionnaire was not considered as valid. Therefore, I have no opinion or I don’t treat children with DS was always mentioned as a possible answer.

Data were collected in a database using an Excel 2010 file. Findings were analysed with SPSS statistics 24. Data were analysed and descriptive parameters were obtained. Cross-tabs and chi-square tests were used to test the correlation between different variables. Results were assessed in 95% confidence interval with *p*< 0.05 level.

## Results

Of the 589 questionnaires that were distributed, 356 were collected and accepted (177 men, 179 women/ response rate 60%). The latter covers 5% of the dentist population in Belgium (7095 working dentists in 2013) (12).

Mean age of the dentists was 50.3 years old (SD: 11.9) and 75% obtained their degree in general dentistry more than 20 years ago. Of all the dentists, 73 (20%) obtained an additional degree but only 6 dentists obtained an additional degree in paediatric dentistry. The majority of the participants work in a dental practice in an urban (city or municipal district) environment (66%), the other part of the dentist (34%) is working in a dental practice in a rural environment.

**Figure 1:**
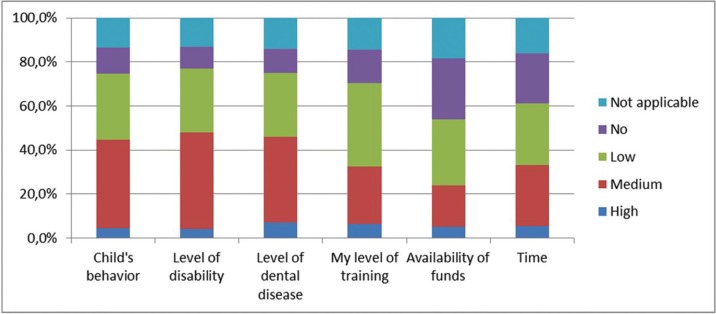
Level of possible barriers for dentists.

The majority of the dentists (78.5%) rarely or never treat a child with DS ( Table 1). Although 49% of the Belgian dentists feel confident enough to treat children with DS, only 14.5% of them think that a general dentist should treat a child with DS and 42.5% refer the child to a specialised centre in a (University) Hospital ( Table 1). Dentists would give themselves a mean score of 4.7 out of 10 for their ability to treat children with DS and in their opinion the highest barriers would be the level of disability (49%), the level of dental disease (48%) and the behaviour of the child (44.5%) (Fig. 1).

**Table 1:**
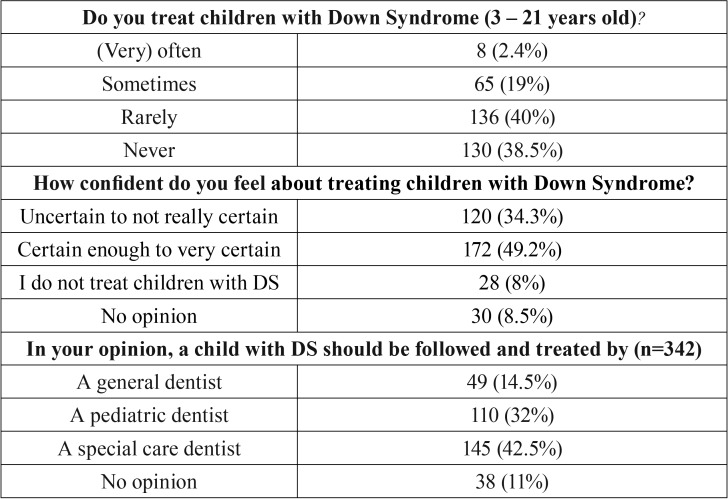
Study respondent characteristics by percentage in different topics.

The accessibility of the practice and the waiting room for children with DS was given a mean score of 7.2 and 7.4 out of 10 respectively. In comparison the same score for children with a physical disability was 6.7 and 6.8.

In more than 52% of the cases, dentists who see or treat children with DS, feel confident to give information about oral hygiene, fluoride therapy, the diet, gum disease, sensitive and missing teeth. Whereas 35% or less of those dentists are able to give information about mouth closure, mouth breathing and OSAS or advice concerning drooling, rinsing the mouth and eruption of teeth. 21% of all the dentists answered to that question that they do not treat children with DS. In contrast when dentists were asked to assess their competencies in performing different dental procedures in children with DS, 25% answered they do not treat children with DS.

Of the dentists, 90% or more don’t seem to have a problem undertaking an examination, to remove calculus and polish the teeth or to give oral hygiene instructions. Half of the dentists (53%) feel confident to restore or seal teeth and 40% would extract a tooth.

Local anaesthesia for dental treatment was used at least once by 56% of the dentists. In general, 12% of the dentists occasionally use oral sedation and 6% have access and the ability to use nitrous oxide in the dental office.

**Figure 2:**
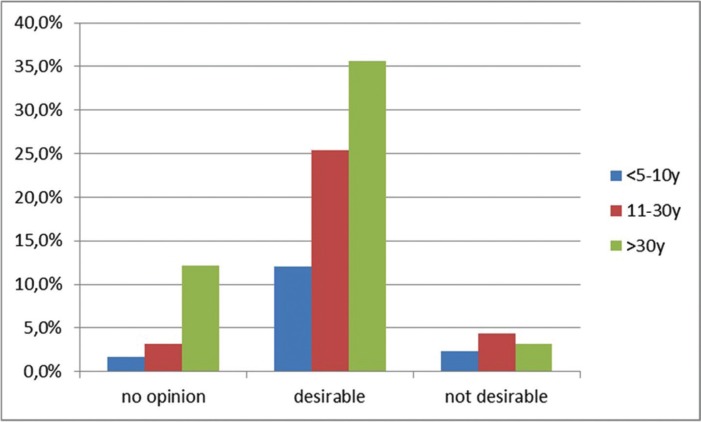
The benefit of obtaining additional training as a function of the age of the dental degree.

A statistically significant association (*p*<0.001) was found between the age of the dental degree (when did the dentist graduate?) and the obtained methods of dental education (did the dentist acquire knowledge concerning the treatment of children with DS?). Younger dentists seem to be more educated in this field compared to the older generation of dentists. There was also a significant association between the age of the dental degree and the possibility of obtaining additional training (*p*<0.01). If a dentist obtained his/her degree more than 10 years ago, there seem to be a lack of knowledge in the field of special care dentistry. In the different age groups of dentists, 72.9% thought that additional training and education would be desirable and 72.5% of all dentists answered that they were not instructed in how to treat children with DS during their undergraduate dental training. Dentists who obtained their degree less than 10 years ago reported that information regarding the care of children with DS was included in the undergraduate program in 62% of the cases whereas only 20% of the older colleagues, who obtained their degree more than 30 years ago, reported to have been prepared to treat children with DS during their undergraduate dental education ( Table 2, Table 3), (Fig. 2).

**Table 2:**
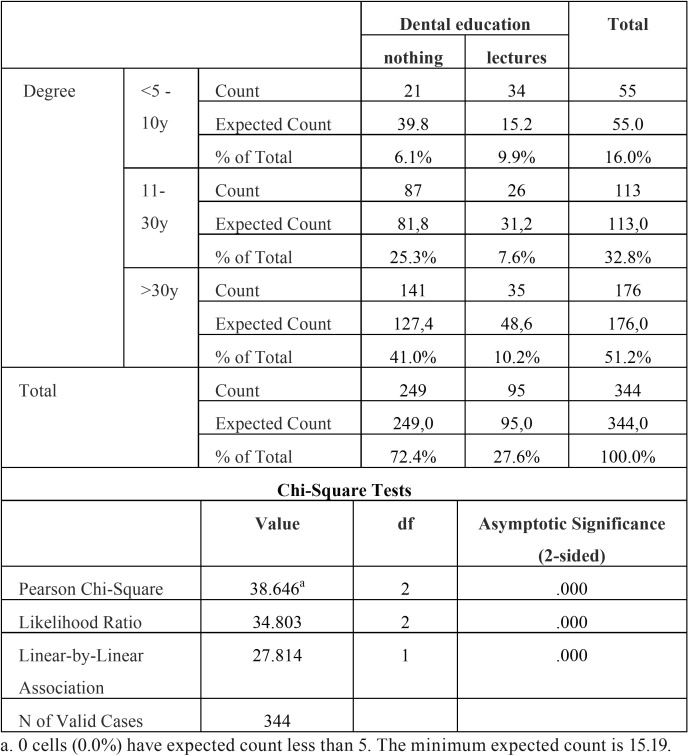
Degree * methods dental education.

**Table 3:**
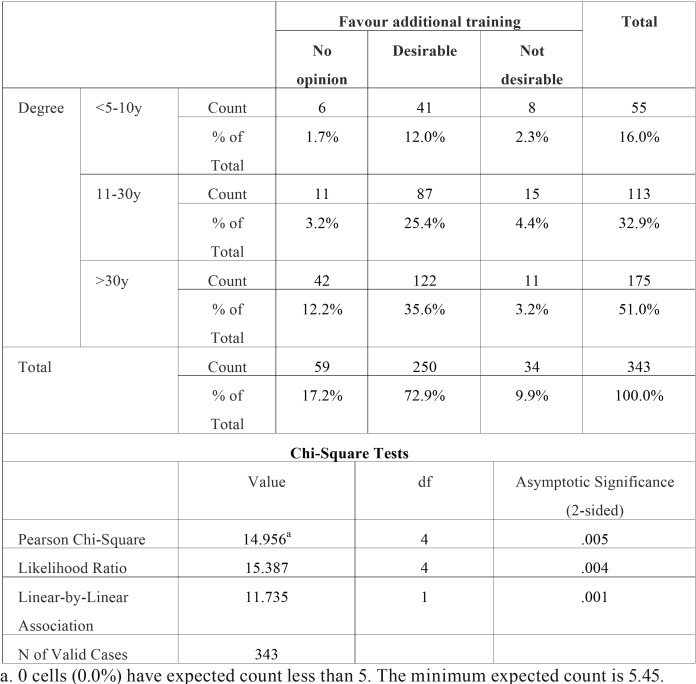
Degree * favour additional training.

## Discussion

To date no similar research to this study has been undertaken in Belgium. Moreover, specific data on the opinion of dentists on the oral health care of children with DS is still scarce in the literature.

The group in this study was representative in that the results cover replies from 5% of the population of Belgian dentists, taking into account a balanced gender and age distribution. Moreover, an even distribution from both parts of the country, in the 2 official languages, was available. The geographical distribution was also varied (34% of the dentists are working in a practice in a rural environment whereas 32% are working in a city).

In the present study almost half of the dentists (49%) stated that they were confident enough to treat children with DS but only 14.5% think that the most suitable dentist to do so is a general dentist ( Table 1). In a previous report, where parents were asked about their views on dental care for their child with DS (10), more than 50% replied that they took their child to a private dental practice, and 53% of the children went to see the same dentist as their sibling(s). This discrepancy shows that parents are more often in favour of attending a general dentist whereas general dentists possibly think that they should transfer the child to a specialized centre in a hospital for the appropriate care. Dentists seem confident to give information about oral hygiene, fluoridation and agenesis and feel confident to perform an examination, to remove calculus and/or polish and to give oral hygiene instructions. This supports the earlier findings in the study of Descamps and Marks (2015) where parents replied that the topics mentioned by the dentist, and the treatments that were performed the most, are the same as those recorded here (11). In the present study 6% of the dentists have the facility to use nitrous oxide in the dental office but none of the parents mentioned that it was used for their child. This is possibly due to the fact that there is still limited consensus in Belgian law to permit dentists to use the system in a private practice and anatomically it’s not possible to always use the system (i.e. extreme mouth breathing, fear of the mask,…) (13).

Dentists don’t seem to have problems dealing with the subjects that are not really specific for children with DS but the barriers to give more specific information and undertaking treatment are still high.

Weil *et al.* collected data from 500 dentists of the MDA (Michigan Dental Association) and the AAPD (American Association for Paediatric Dentistry) to assess educational experience regarding the provision of care for patients with special needs, mental retardation, and autism. General dentists (71%) reported that they had not been sufficiently well prepared to treat patients with mental retardation during their predoctoral education. These results are in line with the results in the present study (72.5%) (9).

In a broader non-DS specific study Casamassimo *et al.* asked 4970 general dentists of the ADA (American Dental Association) chosen at random in 9 different regions to fill in a questionnaire concerning their practice patterns with CSHCN (Children with specific health care needs: cerebral palsy, mental retardation and medically compromised children). Dentists rarely or never treat children with mental retardation in 52% of the cases and 41% of them would desire more training (8). In the present study 78.5% rarely or never treat children with DS and almost 73% of the dentists would desire additional training. Fewer than one in ten general dentists (8.6%) in Casamassimo *et al´s* study often encounter children with special needs in their dental practice whereas in the present study this is only one in twenty.

It’s remarkable that these numbers are still high and it confirms that health care, including access to dental care, is still difficult for this population (8). This is in contrast to the fact that specific regulations and extra financial resources exist in the National Health Insurance system to cover the cost for extra need of care.

Although the dental education program has been changed during the last decade (there has been an increase in time allocated in dental education focussing on the treatment of the special needs population) it is useful to monitor the program for new strategies to educate and improve competencies of (student) dentists to deliver care to all patients with special needs (14).

Kleinert *et al.* tested an interactional virtual patient case, of a ten-year-old-boy with DS, among 50 third year dental students. These students had to make decisions about interaction and clinical procedures and also perform a pre- and post-test for difficulty and knowledge. It is sometimes difficult to provide dental students with clinical experiences concerning treating patients with special needs because of the smaller patient group and therefore an additional computer based program could be useful as a complement to the clinical training (15).

Mac Giolla Phadraig *et al.* also stated that an attitude can change a behaviour so that it is important to improve and change the educational program of undergraduates and thereby try to modify their attitudes (16).

The study group was not small but it must be recognised that there are some limitations to the 

data gathered in the present study. The mean age of the dentists who filled in the questionnaire is about 5 years younger than the mean age of the total population of the Belgian dentists. This can be linked to the fact that the questionnaires were distributed during a continuing education platform. Questionnaires were anonymous in order to avoid bias. Data on children with DS are almost always included in mixed study groups. That is the reason why results of the present study sometimes have been compared to other results concerning patients with mental retardation. Prevention is a must in dentistry and dentists don’t seem to have problems with it. But when a dental problem occurs, the majority of the dentists seem to feel some barrier(s) to treat children with DS.

Only one in four dentists seem to have had any kind of instruction or training during their educational program and the vast majority were in favour of getting additional training. We must be aware that it is not because students or dentists receive complementary training, that they will automatically treat more patients with special needs. This depends on the attitude of people, combined with the financial and time aspect (16).

It is however positive that the undergraduate program has been changed in the last decade and dentists are in favour of getting additional training and education to help them improve their knowledge of, and to feel more confident in, treating children with DS, or even broader patients with special needs, in daily practice.
